# Enhancing underwater target localization through proximity-driven recurrent neural networks

**DOI:** 10.1016/j.heliyon.2024.e28725

**Published:** 2024-03-30

**Authors:** Sathish Kumar, Ravikumar Chinthaginjala, Dhanamjayulu C, Tai-hoon Kim, Mohammed Abbas, Giovanni Pau, Nava Bharath Reddy, Member, IEEE

**Affiliations:** a School of Electronics Engineering, Vellore Institute of Technology, Vellore, 632014, India; b School of Electrical and Computer Engineering, Yeosu Campus, Chonnam National University, 50 Daehak-ro, Yeosu-si, Jeollanam-do, 59626, Republic of Korea; c Electrical Engineering Department, College of Engineering, King Khalid University, Abha, 61421, Saudi Arabia; d Faculty of Engineering and Architecture, Kore University of Enna, 94100, Enna, Italy

**Keywords:** Localization, RNN, UWSN, Distance, Angle, Mean estimation error

## Abstract

Environmental monitoring, ocean research, and underwater exploration are just a few of the marine applications that require precise underwater target localization. This study goes into the field of underwater target localization using Recurrent Neural Networks (RNNs) enhanced with proximity-based approaches, with a focus on mean estimation error as a performance metric. In complex and dynamic underwater environments, conventional localization systems frequently face challenges such as signal degradation, noise interference, and unstable hydrodynamic conditions. This paper presents a novel approach to employing RNNs to increase the accuracy of underwater target localization by exploiting the temporal dynamics of proximity-informed data. This method uses an RNN architecture to track changes in audio emissions from underwater targets sensed by a microphone network. Using the temporal correlations represented in the data, the RNN learns patterns indicative of target localization quickly and correctly. Furthermore, the addition of proximity-based features increases the model's ability to understand the relative distances between hydrophone nodes and the target, resulting in more accurate localization estimates. To evaluate the suggested methodology, thorough simulations and practical experiments were carried out in a variety of underwater environments. The results show that the RNN-based strategy beats conventional methods and works effectively even in difficult settings. The utility of the proximity-aware RNN model is demonstrated, in particular, by considerable reductions in the mean estimate error (MEE), an important performance measure.

## 1 Introduction

Underwater wireless sensor networks (UWSNs) present their own set of unique challenges due to factors such as water fluidity, the complexity of the nodes' three-dimensional spatial arrangement, the difficulty of synchronizing the clocks of underwater nodes, and the large errors introduced by ranging algorithms influenced by water flow [1]. A solution is required because positioning algorithms in UWSNs are vulnerable to unstable node placements and severe positioning difficulties as a result of these causes [2]. A non-located node can filter the received signal intensity from anchor nodes and then apply a weighted correction to limit the harmful effects of the aquatic environment on measurement precision. The use of geolocation services is extremely important for underwater communication systems in order to get information about their surroundings [3]. In recent years, the use of underwater acoustic sensor networks, often known as UWSNs for short, has increased in popularity as a result of the numerous advantages they offer for ocean research and monitoring. More significantly, acoustic communications have increasingly superseded radio frequency transmissions as the predominant means of communication, thereby establishing their pre-eminence in this field. This has helped acoustic communications cement its supremacy. This is extremely important due to the fact that radio frequency broadcasts were the primary form of communication for a considerable amount of time [4].

Many fields rely on precise target localization in the context of underwater exploration and surveillance, including marine biology research, environmental monitoring, resource discovery, and underwater robots [[5], [6], [7]]. Underwater settings present distinct challenges in terms of signal transmission, noise, and hydrodynamic complexity, all of which necessitate creative ways to enhance target localization accuracy and dependability. This study introduces "Cognitive Localization (Cogni Loc)," a revolutionary way of overcoming these constraints that uses Proximity-Driven Recurrent Neural Networks to locate the exact location of items in the water. Traditional localization methods usually yield erroneous results due to the intricacy of underwater surroundings. The proposed method takes advantage of the temporal patterns inherent in proximity-based data to significantly improve the precision with which targets can be located in underwater environments [8].

RNNs have gained popularity in recent years due to their capacity to represent sequential data and find temporal correlations and patterns. In the context of underwater target localization, these networks are used to learn about the dynamic properties of acoustic signals emitted by underwater targets and picked up by hydrophone arrays [9,10]. Because they are trained to account for the changing character of acoustic signatures over time, RNNs can distinguish underlying patterns that serve as useful indications for target localization. The relative distances between the hydrophone nodes and the target are also used as contextual information in this novel method. These properties increase the model's target location estimation accuracy significantly by providing critical information about the target's spatial arrangement [[11], [12], [13]].

Extensive simulations and real-world investigations were conducted in a wide range of underwater environments to validate Cogni Loc's efficacy. The results show that the proposed method outperforms state-of-the-art alternatives, demonstrating its durability and flexibility even in tough settings such as signal degradation and changing environmental conditions [14]. As demonstrated in the following portions of this study, we then dive into the intricacies of the Cogni Loc framework, detailing its design, data processing pipeline, and implementation of proximity-driven features. We also present a detailed analysis of the results of the studies, commenting on how the combination of RNNs with proximity-based approaches improved target localization accuracy and revealed additional advantages [15]. This research has far-reaching implications for underwater navigation, exploration, and monitoring in the future, opening up new avenues for better understanding and exploiting the ocean's unseen landscapes.

In the context of enhancing RNNs, proximity-based strategies involve incorporating spatial or temporal correlations between data points into the network design to improve its understanding and prediction capabilities. These strategies can take a variety of approaches, such as including proximity metrics between input sequences or using attention mechanisms to focus on neighboring parts within the data sequence. For example, proximity information can be integrated by adding new features that represent spatial or temporal links between data points, allowing the RNN to capture dependencies more efficiently. Furthermore, techniques such as spatial or temporal attention mechanisms allow the model to assign varying levels of importance to different elements based on their proximity or relevance to the current context, enhancing the model's ability to detect critical information within the input sequence. RNNs can better capture the underlying structure and patterns in sequential data by using proximity-based methodologies, resulting in enhanced performance in applications like sequence prediction, language modelling, and time-series analysis.

A. Problem statement

Underwater target localization remains a key difficulty in underwater exploration, de-fence, and environmental monitoring applications due to the complexity of the underwater environment. Significant errors in localization can be attributed to a variety of problems, including water fluidity, three-dimensional sensor node structure, a lack of dependable clock synchronisation, and the disruptive impact of water flow on range algorithms. Because existing approaches have trouble providing precise and consistent data, underwater wireless sensor networks have limited performance and utility. These issues need the development of a novel method for improving the accuracy and reliability of underwater target localization.

B. Motivation

The widespread use of UWSNs in a wide range of mission-critical applications, as well as the inherent limitations associated with underwater target localization, drive the investigation of cutting-edge techniques to address these challenges. Existing methodologies often fail to give precise and trustworthy localization findings due to the ocean's fluid and unpredictable character [16]. As a result, we must adopt more cutting-edge ways that make use of today's technology breakthroughs.

An approach designated "Enhancing Underwater Target Localization through Proximity-Driven Recurrent Neural Networks" has been presented to improve underwater target localization. First and foremost, the dynamic character of underwater acoustic waves emitted by targets and sensed by hydrophone arrays can be represented using Recurrent Neural Networks, which have been found to be effective in simulating temporal relationships within sequential data. Second, by incorporating proximity-based techniques, localization accuracy can be considerably enhanced by taking into account the relative distances between hydrophone nodes and targets [17,18].

By combining the capabilities of RNNs with proximity-based information, this technique aims to revolutionize underwater target localization. The desire to achieve new levels of precision and dependability in a field where more traditional approaches have failed serves as motivation. This method could have a wide range of applications, from marine research and conservation to environmental protection and underwater robots. The ultimate goal is to find new ways to understand and exploit the latent dynamics of the marine domain.

C. Contributions

## 2 The following are the manuscript's most significant contributions

➢ The primary contribution of the approach lies in its ability to enhance the accuracy of underwater target localization. By leveraging the temporal patterns embedded in acoustic signals through Recurrent Neural Networks (RNNs), and incorporating proximity-based features, the proposed method surpasses traditional techniques in accurately estimating the positions of underwater targets.
➢ The approach addresses the challenges posed by the dynamic and unpredictable underwater environment by the integration of proximity-based information and RNNs enabling the model to better adapt to these challenges, resulting in improved performance even under adverse conditions.
➢ The combination of RNNs and proximity-based techniques represents a novel technological innovation in the realm of underwater target localization. This approach showcases the potential for modern machine learning and sensor fusion techniques to revolutionize the capabilities of underwater sensor networks.
➢ The advancements brought about by this approach have potential implications beyond underwater target localization. The integration of proximity-driven techniques with neural networks could find applications in various domains involving localization and spatial awareness.

The manuscript is organized as follows: Section 2 goes over the relevant works. The suggested network design and simulation settings of the underwater localization methods are discussed in Section 3. The fourth section examines the simulation results. Section 5 begins with a plan summary, followed by a series of final observations and future scope.

## 3 Literature survey

Cao, X et al. [19], developed a tracking control system for autonomous underwater vehicles. This solution will employ trajectory prediction to handle the difficulty of tracking dynamic targets while submerged. To begin, we are designing a target detection system that takes advantage of deep learning. In this method, the YOLO v3 network is used to determine the location of the target within a sonar image acquired from a multibeam-forward-looking sonar system. Following that, we build a temporal profit Elman neural network, also known as a TPENN, to forecast the trajectory of the moving item. When compared to a traditional Elman neural network, its dynamic target prediction accuracy is significantly higher. Using the model predictive controller (MPC), we are able to properly track a moving item while it is submerged in water, and the results of our tracking are both dependable and consistent. It was proved through modelling and experimentation that the strategy presented for directing the tracking of dynamic targets underwater is both effective and practicable.

Song D et al. [20], investigated the application of deep reinforcement learning, often known as DRL, for target tracking within the context of multiple autonomous surfaces underwater vehicle guidance and control architecture. The framework instructs the vehicles to accomplish the standoff tracking and sampling tasks along a circular route centered on the target while maintaining present relative positions throughout the procedure. This enables the collection of precise spatiotemporal synchronisation data. We can design autonomous systems that are not sensitive to their aims or environments by establishing an end-to-end framework that maps sensor inputs to control commands. As a result, co-operative guiding, standoff tracking, and dynamic obstacle avoidance are all made possible. The data show that the end-to-end DRL technique is effective. The end-to-end DRL method is more precise than the traditional two-stage "guidance-control" procedure. In the interim, additional funding is supplied for the experiment on obstacle avoidance and standoff tracking for the swarm, as well as the experiment on sampling in the mesoscale eddy area, to ensure the proposed framework's efficiency and resilience.

Wang, M et al. [21], started by learning about the qualities that the majority of underwater targets held, and then we built the target dataset to meet these specifications. Second, we created a CNN model to find the target and assess the utility of tracking a moving object. We first tweaked the Kalman filter and then used an artificial neural network with both long-term and short-term memory (LSTM-NN) to update it regularly. This enabled us to create more precise projections of the target's azimuth and distance. Following that, we tested the novel method using both simulations and actual data from an acoustic sea trial to validate it. The results of the simulation testing revealed that the relative error of the LSTM-Kalman filtering technique was reduced by 60%, and by 72.25% in the sea trial, with an estimation variance of only 4.79. The technique created for determining the positions of underwater targets is both effective and accurate, according to the findings of this study.

Shin, W et al. [22], proposed a deep learning-based segmentation network for BTR images to improve opponent recognition accuracy in marine scenarios. This will enable more precise identification of possible adversaries. We relied on the spatial convolutional layer to extract the elements we wanted. Furthermore, we present unique loss functions for network training in order to address the major class imbalance problem in BTR images. Furthermore, we overcame the issue of obtaining precise target-bearing data for military reasons by creating a synthetic BTR dataset that replicates the diverse underwater environments. As a workaround, this was done. We conducted extensive testing and in-depth talks using our synthetic BTR dataset to demonstrate that the proposed network outperforms state-of-the-art approaches for target segmentation.

Nie, W et al. [23], proposed a model's input in the first phase features local beam patterns that have been augmented utilizing a number of data augmentation methodologies. Each of these patterns contains critical information that can be utilised to detect targets. In the second stage, you should think about creating a classification model with the help of an adaptive convolutional neural network (CNN). It is feasible to train models using information from a single source and then test those models using information from several sources. The concept is suitable for arrays of hydrophones with a wide range of geometries and numbers. State-of-the-art DOA estimate methods are used to assess the effectiveness of the proposed strategy. Conventional beamforming (CBF), multiple signal classification (MUSIC), minimum-variance distortion less response (MVDR), and sparse Bayesian learning (SBL) are among these approaches. The proposed method outperforms three simulated situations and two sets of recorded data from various marine locations in terms of directional accuracy and angular root-mean-squared error (RMSE).

He et al. [24], described a novel strategy to improving energy economy and extending the lifespan of Underwater Wireless Sensor Networks (UWSNs) by offering an energy-efficient clustering and multi-hop routing protocol based on metaheuristics. Unlike existing techniques that use separate algorithms for clustering and multi-hop routing, which increases computational complexity and complicates parameter adjustment, the proposed hierarchical chimp optimization (HChOA) framework combines the two processes. HChOA's effectiveness is shown through thorough simulations and metric evaluations. Comparative analyses against recognized protocols such as LEACH, TEEN, MPSO, PSO, and IPSO-GWO demonstrate HChOA's superiority in terms of UWSN lifespan extension and energy conservation, confirming its potential for improving underwater sensor network efficiency.

Yang et al. [25], proposed the use of the Chimp Optimization Algorithm (ChOA) to choose cluster heads and effectively organize clusters, followed by the Hierarchical Greedy Search (HGS)-based routing strategy to discover optimal pathways within the network. The proposed ChOA-HGS approach attempts to maximize network lifetime while also increasing energy efficiency in a synergistic manner by integrating clustering and routing functionalities. The ChOA-HGS framework is validated by extensive simulations across three different situations, with performance measured using a variety of indicators. A comparison with established protocols such as PSO, MPSO, IPSO-GWO, TEEN, and LEACH demonstrates ChOA-HGS' superiority in terms of network longevity and energy consumption efficiency, confirming its effectiveness as a promising solution for improving the performance of wireless sensor networks.

Khishe et al. [26], introduced a novel underwater acoustic target recognition model called Attention Mechanism Residual Concatenate Network (ARescat), which combines residual concatenate networks with Squeeze-Excitation (SE) attention mechanisms and uses joint supervision with Focal Loss for precise feature classification. Using recognition experiments on the ShipsEar database, the paper compares the performance of the ARescat model to the standard ResNet18 model under identical feature extraction settings. The results show that the ARescat model, despite having the same amount of model parameters as ResNet18, has a 2.8% higher recognition accuracy, reaching an astonishing 95.8%. This improvement is especially noteworthy when comparing other models and feature extraction approaches, demonstrating the ARescat model's superior performance in underwater acoustic target recognition tasks.

Khishe et al. [27], proposed the use of the Moth Flame Optimization (MFO) algorithm to fine-tune Deep Neural Networks for recognizing various underwater sonar datasets, while acknowledging the common challenges that metaheuristic algorithms face, such as premature convergence, local minima entrapment, and failure to converge within reasonable timeframes, particularly in high-dimensional search spaces. It emphasizes the critical significance of spiral flight within the MFO, which determines how moths modify their positions relative to flames, managing the transition between the exploration and exploitation stages. The work analyses seven spiral motions with different curvatures and slopes to improve MFO performance, notably in underwater target classification tasks, using the benchmark Sejnowski & Gorman dataset and two experimental sonar datasets: passive and active. Comparative analyses against four nature-inspired algorithms (Heap-Based Optimizer, Chimp Optimization Algorithm, Ant Lion Optimization, Stochastic Fractals Search) and Particle Swarm Optimization confirm the customized MFO's superior performance, as evidenced by increased classification rates of 1.5979, 0.9985, and 2.0879 for Sejnowski & Gorman, passive, and active datasets, respectively, without significant increases in time complexity, highlighting its efficacy.

## 4 Network architecture and modelling

To improve underwater target localization inside a UWSN, the proposed network design integrates improved Received Signal Strength Indicator (RSSI) measurements and the usage of Recurrent Neural Networks (RNNs). This simulation system employs a redesigned RSSI measurement model to simulate the complicated dynamics of signal propagation, noise, and interference found underwater [28]. We create synthetic UWSN data that more closely resembles real-world UWSN conditions by including anchor node positions, target positions, and improved RSSI values. Throughout the training process, which is carried out in a sequence-to-sequence way, the better RSSI measurements are used as inputs and the desired locations are obtained as outputs. Among the simulation parameters are anchor node density, UWSN dimensions, path loss characteristics, and training iterations. The RNN is evaluated using two assessment metrics: mean squared error and localization accuracy. This comprehensive solution is intended to address the special challenges of UWSNs in a wide range of aquatic environments, and it shows promise in significantly improving underwater target localization accuracy by syncing advanced RNN architectures with increased RSSI data.

The distance-based localization technique is implemented after a thorough investigation of a 120-m square network field within an underwater environment. This is accomplished through the use of MATLAB, which ensures a smooth workflow. The primary goal of this phase is to assess the effect of distance on localization accuracy [29]. Four anchor nodes strategically placed at the network's cardinal points help determine an object's relative position in space. The network field itself is a 120-m square in this context, containing ten mobile nodes, one of which is assigned as the target for tracking. The target's starting location is chosen at random, and recurrent trials are used to refine its position. It's worth noting that only a portion of these trials are taken into account in this scenario [30].

**Fig. 1:**
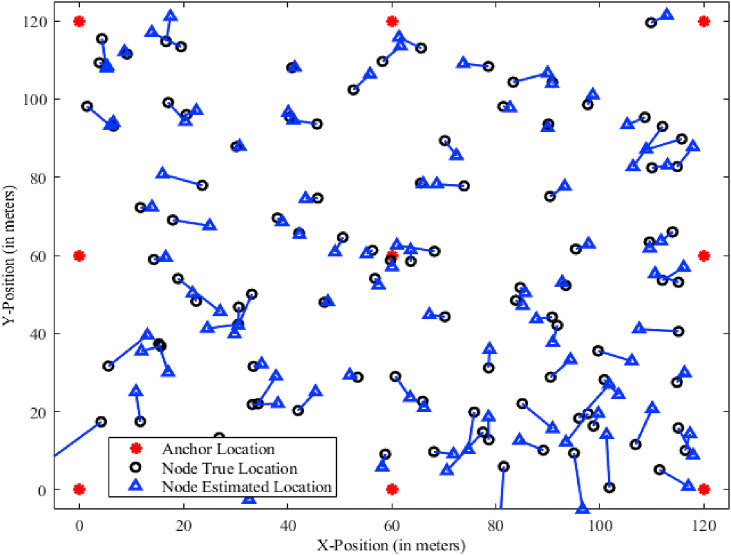
RNN - UWSN node distribution.

**Fig. 2:**
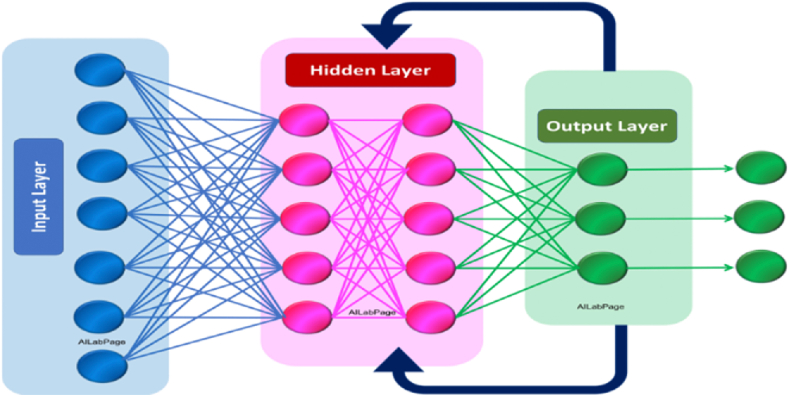
A typical architecture of RNN – UWSN.

Data from an initial set of 8 trials is used to estimate the mobile node's position. The beacon sensor nodes, which are linked to reference antennas, make it easier to calculate distances between mobile sensor nodes and associated beacon nodes [31]. This distance information is used to compute the distances between vertices. Fig. 1 depicts the node distribution inside the UWSN, illustrating the spatial layout of sensor nodes and anchor nodes across the 120-m square network area. This scenario sheds light on the relationship between distance and localization accuracy, paving the door for further investigation and system optimization as shown in Fig. 2.

D. Feedforward Calculation

The feedforward process computes the output of each layer in the neural network. Assuming we have an input vector x and a DNN with L layers as in Equations (1), (2) [32]:

(1) $$z^{\left(l\right)}=b^{\left(l\right)}{+W}^{\left(l\right)}a^{\left(l-1\right)}$$

(2) $$a^{\left(l\right)}=z^{\left(l\right)}\sigma$$

Where $z^{\left(l\right)}$ weighted sum at layer *l*.

$W^{\left(l\right)}$ Weight matrix at layer *l*.

$a^{\left(l-1\right)}$ Output of the previous layer.

${\;b}^{\left(l\right)}$ Bias vector at layer *l*.

σ Activation function.

E. Loss Function

The loss function measures the discrepancy between the predicted output and the actual target. For regression tasks, MEE is commonly used Equation (3):

(3) $$MEE=\frac{1}{N}\sum_{i=1}^{N}{\left(y_{i}-y_{i}^{ˆ}\right)}^{2}$$

Where.

N number of samples.

$y_{i}$ True target value for the ith sample.

$y_{i}^{ˆ}$ Predicted value for the ith sample.

F. Backpropagation

Backpropagation computes the gradients of the loss with respect to the network's parameters, allowing us to update the parameters during training Equation (4) [33]:

(4) $$\frac{\partial MEE}{{\partial W}^{\left(l\right)}}=\frac{2}{N}\;\sum_{i=1}^{N}\left(y_{i}-y_{i}^{ˆ}\right).\sigma^{\prime }\left(Z^{\left(L\right)}\right).a^{\left(L-1\right)}$$

*L* output layer index.

$\sigma^{\prime }\left(.\right)$ Derivative of the activation function.

$a^{\left(L-1\right)}$ Output of the previous layer.

G. Gradient Descent Update

The gradient descent update rule adjusts the weights and biases using the computed gradients Equations (5), (6)*:*

(5) $$W^{\left(l\right)}=W^{\left(l\right)}-\alpha \frac{\partial MEE}{{\partial W}^{\left(l\right)}}$$

(6) $$b^{\left(l\right)}=b^{\left(l\right)}-\alpha \frac{\partial MEE}{{\partial b}^{\left(l\right)}}$$

Where:

*l* is the layer index.

*α* is the learning rate.

H. Regularization

Regularization techniques such as L2 regularization Equation (7) can be applied to prevent overfitting [34,35]:

(7) $$L_{reg}=\frac{\lambda }{2N}\;\sum_{l=1}^{L}{\left\|W^{\left(l\right)}\right\|}_{2}^{2}$$

Where *λ* is the regularization strength.

*N* is the number of samples.

Because of its capacity to capture temporal connections in sequential data, an RNN architecture is well suited for applications such as underwater target localization within UWSNs [[36], [37], [38]]. This is because RNNs are capable of doing so. The method incorporates sequence intake, temporal pattern extraction, and outcome prediction across three layers: input, recurrent, and output [39]. At each time step, the input layer will accept data sequences, and the RSSI readings collected from anchor nodes will be of particular interest. To record hidden states throughout time, an equation incorporating hyperbolic tangent activations and weight matrices is utilised, along with the linkages that connect the input to hidden states and hidden states to other hidden states that are related to those states [[40], [41], [42], [43]]. The output layer can create forecasts by using the hidden state as well as a softmax operation to standardize the probability distribution. The input weight matrices and bias terms are used to make these predictions. RNN perform localization tasks and comprehend underlying patterning in this configuration [44]. RNN applied to UWSN, along with the corresponding mathematical equations from (8), (9), (10).

I. RNN Architecture for UWSN

An RNN is designed to capture temporal dependencies in sequential data, which makes it suitable for tasks like underwater target localization in UWSNs. The architecture involves an input layer, a recurrent layer, and an output layer.

**Input Layer:** Takes sequences of data as input at each time step.

**Recurrent Layer:** Contains recurrent units that maintain hidden states to capture temporal patterns.

**Output Layer:** Produces the final output prediction based on the learned temporal dependencies.

Assuming a simple RNN cell, the mathematical equations for each component are as follows:

Input Layer:

At each time step t, the input to the RNN cell is the sequence of data from anchor nodes as in Equation (8) (e.g., RSSI measurements):

(8) $$X_{t}={\left[x_{1,t};x_{2,t};\ldots \ldots \ldots \ldots .x_{N,t}\right]}^{T}$$

Where $x_{i,t}$ is the data from the ith anchor node at time t.

Recurrent Layer (Simple RNN Cell):

The recurrent layer contains a hidden state $h_{t}$ that evolves over time based on the input and the previous hidden state. Here's the basic update equation for a simple RNN cell Equation (9):

(9) $$h_{t}=\tanh\;\left(W_{hx}h_{t}+W_{hh}h_{t-1}+b_{h}\right)$$

${\text{Where}\;W}_{hx}$ Weight matrix for the input-to-hidden connection.

${\;b}_{h}$ Bias term.

tanh Hyperbolic tangent activation function.

${\;W}_{hh}$ Weight matrix for the hidden-to-hidden connection.

${\;h}_{t}$ Hidden state at time t.

**Output Layer:** The output at each time step t can be calculated using the hidden state $h_{t}$ as in Equation (10)

(10) $$y_{t}=\text{softmax}\;\left({h_{t}W}_{\text{hy}}+b_{y}\right)$$

Where softmax is used to normalize the output into a probability distribution, and $W_{\text{hy}}$ and $b_{y}$ are the weight matrix and bias term for the output layer.

**Table:** **Algorithm:** Proximity-Driven Recurrent Neural Networks for UWSN algorithm

Input Layer: $X_{t}={\left[x_{1,t};x_{2,t};\ldots \ldots \ldots \ldots .x_{N,t}\right]}^{T}$
Recurrent Layer: (Simple RNN Cell): $h_{t}=\tanh\;\left(W_{hx}h_{t}+W_{hh}h_{t-1}+b_{h}\right)$
Output Layer: $y_{t}=\text{softmax}\;\left({h_{t}W}_{\text{hy}}+b_{y}\right)$
1. Define network parameters (N: number of anchor nodes, input_size, hidden_size, output_size).
2. Initialize weight matrices W_hx_, W_hh_, W_hy_ and bias terms b_h_, b_y_.
3. Set learning rate and other hyper parameters.
4. Define training data: sequences of RSSI measurements and corresponding target positions.
5. for each epoch in range (num_epochs):
6. for each time step t in range (sequence_length):
7. Input data at time step t: $X_{t}={\left[x_{1,t};x_{2,t};\ldots \ldots \ldots \ldots .x_{N,t}\right]}^{T}$
8. Calculate hidden state at time t: $h_{t}=\tanh\;\left(W_{hx}h_{t}+W_{hh}h_{t-1}+b_{h}\right)$
9. Calculate output prediction at time t: $y_{t}=\text{softmax}\;\left({h_{t}W}_{\text{hy}}+b_{y}\right)$
10. Calculate loss using predicted output y_t_ and target position
11. Back propagate gradients through the network
12. Update weights and biases using gradient descent or optimizer
13. End For

**Fig. 3:**
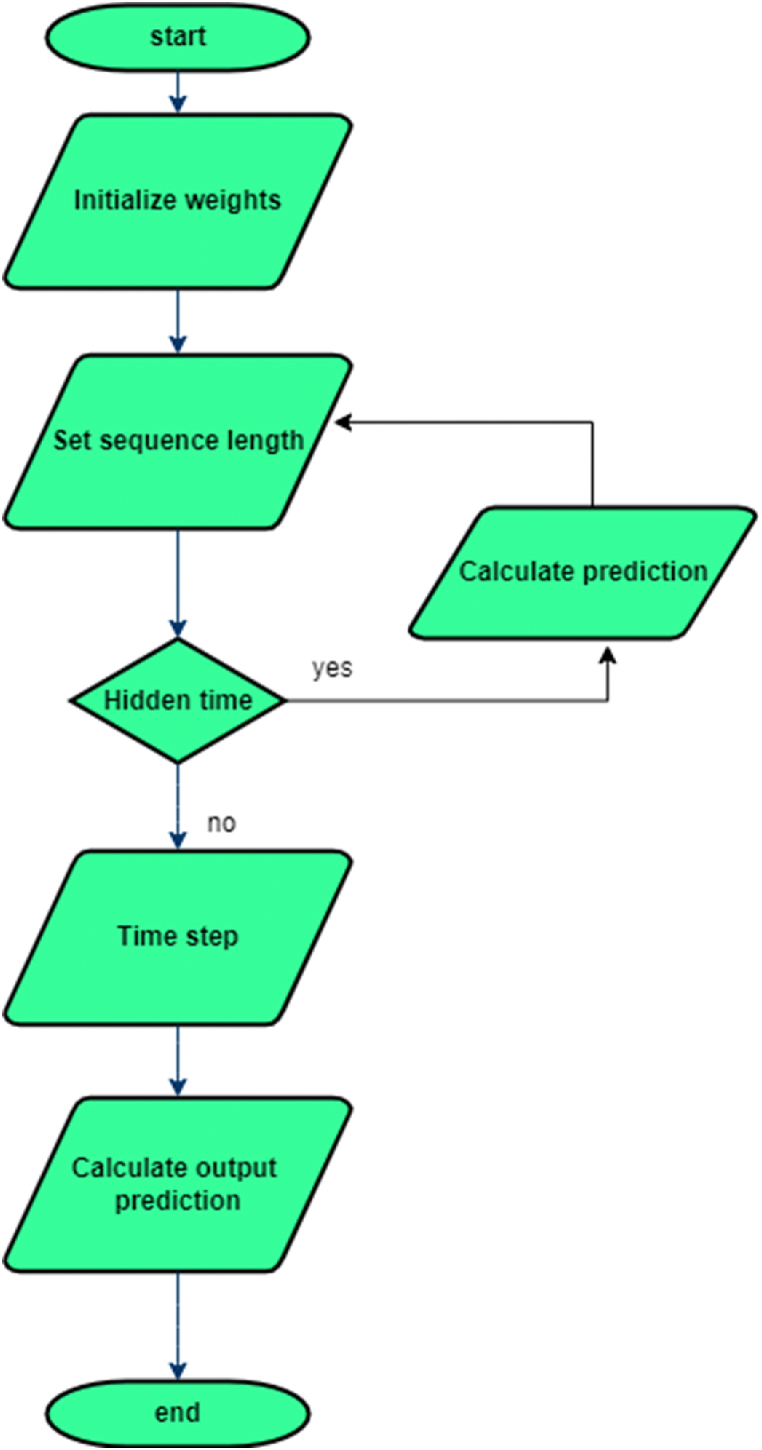
Process flow of RNN – UWSN.

Fig. 3 depicts the steps involved in training a neural network-based localization model utilizing received signal strength indicator values. The number of anchor nodes (N), input size, hidden size, and output size are all characteristics that form the network design. Initialization of weight matrices (W_hx_, W_hh_, W_hy_) and bias terms (b_h_, b_y_). The hyper parameters are configured, and training data consisting of RSSI measurement sequences and matching target positions is defined. The model is trained over a predetermined number of epochs. Within each epoch, input data for each time step in the series is prepared and used to construct a hidden state using activation functions and the preceding hidden state. A softmax function is used to anticipate the output. The network calculates loss by comparing predictions to target positions, and gradients are back-propagated. Optimization techniques are used to update weights and biases. This method is repeated until the number of epochs requested is attained, resulting in a trained model capable of predicting positions based on RSSI readings.

## 5 Simulation results and discussion

The underwater research confirmed the efficacy of the created distance- and angle-based metrics, providing significant confirmation for using the RSSI method in the study titled "Enhancing Underwater Target Localization through Proximity-Driven Recurrent Neural Networks" using MATLAB with simulation area of 120 m * 120 m for various iterations. In this work, the mean estimation error is an important parameter for analysing localization performance. The study analyses accuracy using an RSSI-based efficient localization approach, which quantifies the differences between estimated and ground-truth positions of sensor nodes. Notably, the newly proposed RSSI-based method outperforms baseline techniques in terms of accuracy, as seen by better mean estimation error outcomes. The geolocation algorithm has been improved with efficiency-driven approaches to reduce energy consumption and communication overhead, which has an effect on the standard error of estimates. The research looks on the effects of signal attenuation, multipath fading, and environmental factors on mean estimation error in depth. Recognizing limitations such as signal interference and deployment variability, the study identifies areas for additional investigation and improvements to reduce mean estimation error and improve system reliability. The reduction in mean estimation error demonstrated the method's practical usefulness under underwater settings, with future real-world applications looking for potential.

J. Assessment of MEEs based on TDOA

**Fig. 4:**
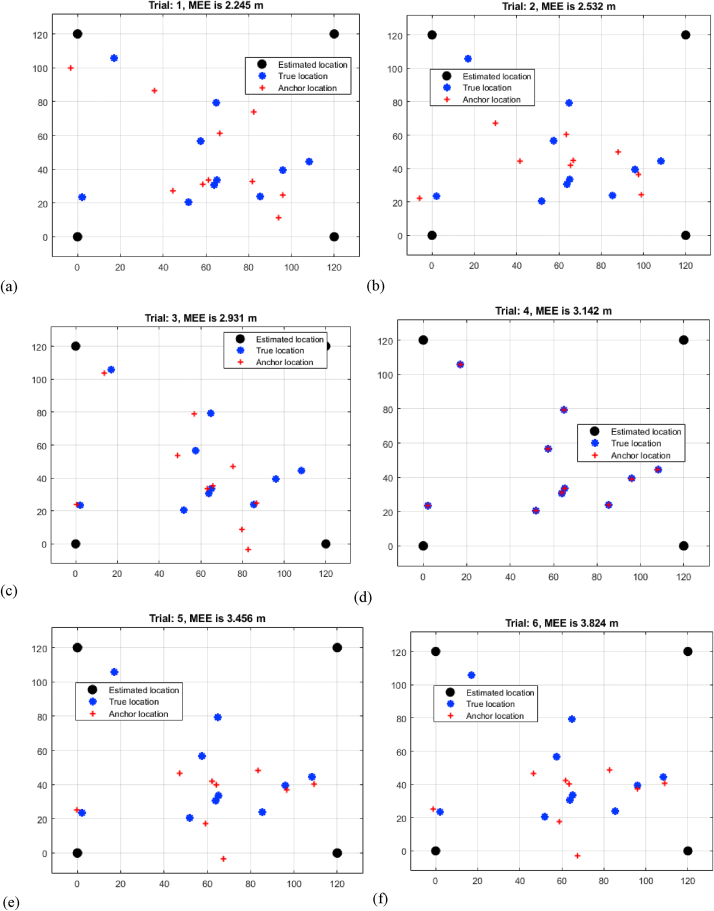

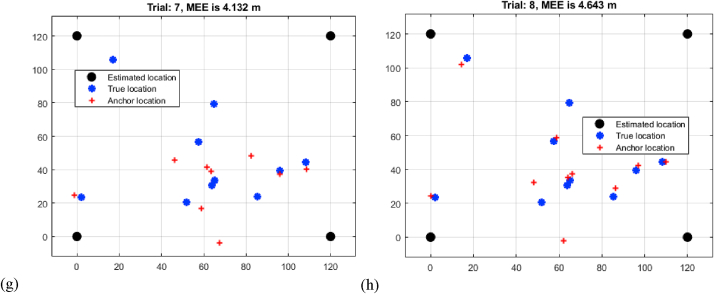
Multi-iteration TDOA mean estimation error analysis (a) trial 1 (b) trial 2 (c) trial 3 (d) trial 4 (e) trial 5 (f) trial 6 (g) trial 7 (h) trial 8.

Table 1's result analysis, headed "Multi-Iteration TDOA Mean Estimation Error Analysis," provides a thorough knowledge of the Mean Estimation Errors (MEEs) produced from Time Difference of Arrival (TDOA) measurements across eight trials of varied distances. As the distance between sensors increases, the observed pattern shows an increase in MEEs. Trial 1 has the lowest MEE of 2.245 m for a shorter distance, while Trial 8 has the greatest MEE of 4.643 m for a longer distance as shown in Fig. 4(a–h). This study highlights the difficulty of accurately calculating target positions at larger distances using TDOA data. The gradual increase in MEEs highlights the influence of signal propagation delays and measurement uncertainties that accrue over time. This trend sheds light on the TDOA method's limitations, implying that further development or the incorporation of additional measurement modalities may be required to improve localization accuracy at greater ranges.

K. Assessment of MEEs based on RSSI

**Table 1:** Multi-iteration TDOA mean estimation error analysis.

Trial	Distance (m)
1	2.245
2	2.532
3	2.931
4	3.142
5	3.456
6	3.824
7	4.132
8	4.643

**Fig. 5:**
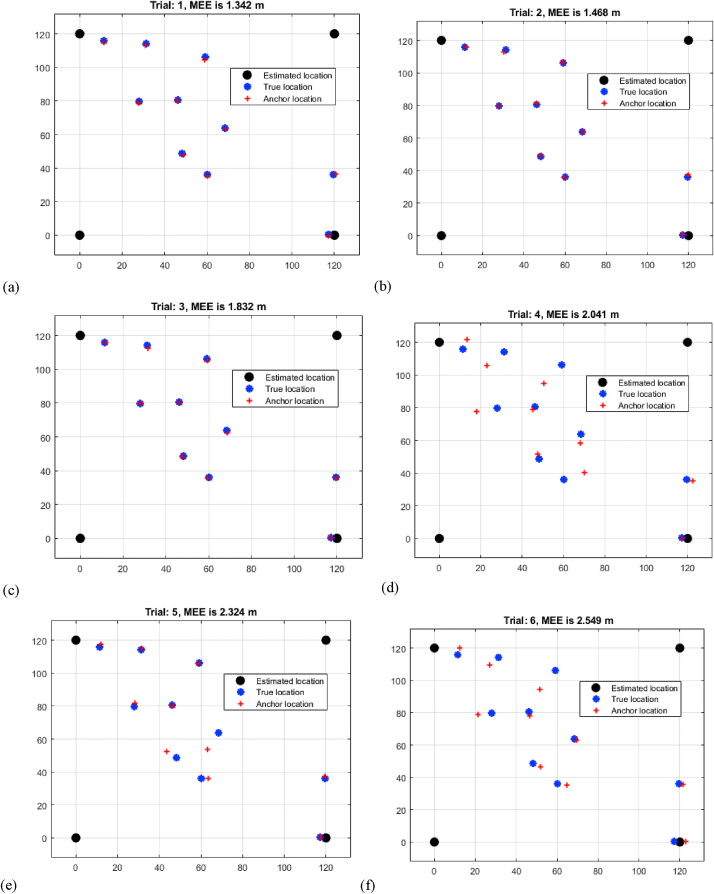

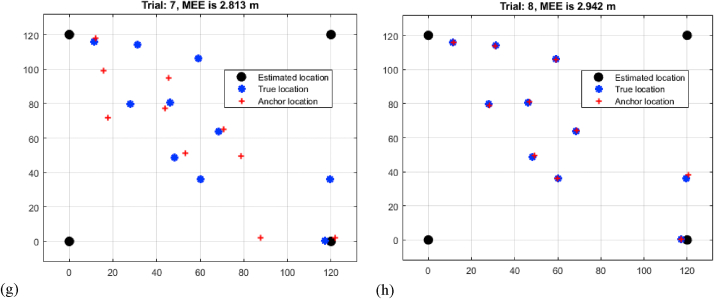
Multi-iteration RSSI mean estimation error analysis (a) trial 1 (b) trial 2 (c) trial 3 (d) trial 4 (e) trial 5 (f) trial 6 (g) trial 7 (h) trial 8.

The study in Table 2, titled "Multi-Iteration RSSI Mean Estimation Error Analysis," provides useful insights into the Mean Estimation Errors (MEEs) produced from Received Signal Strength Indication (RSSI) measurements across eight trials of varied distances. The results show a progressive increase in MEEs as the distance between sensors increases. Trial 1 has the shortest MEE of 1.342 m for a shorter distance, whilst Trial 8 has the greatest MEE of 2.942 m for a longer distance shown in Fig. 5(a–h). This increase in MEEs demonstrates the impact of signal attenuation and interference, which leads to decreased accuracy in determining target positions at long ranges. The results highlight the difficulties involved with RSSI-based localization across larger distances. As MEEs climb gradually, it emphasizes the significance of accounting for signal strength changes and ambient conditions when using RSSI measurements for precise target localization. This analysis highlights the RSSI approach's possible shortcomings and highlights the need for solutions that might offset the negative impacts of signal fluctuation for increased localization precision.

L. Assessment of MEEs based on RNN RSSI

**Table 2:** Multi-iteration RSSI mean estimation error analysis.

Trial	Distance (m)
1	1.342
2	1.468
3	1.832
4	2.041
5	2.324
6	2.549
7	2.813
8	2.942

**Fig. 6:**
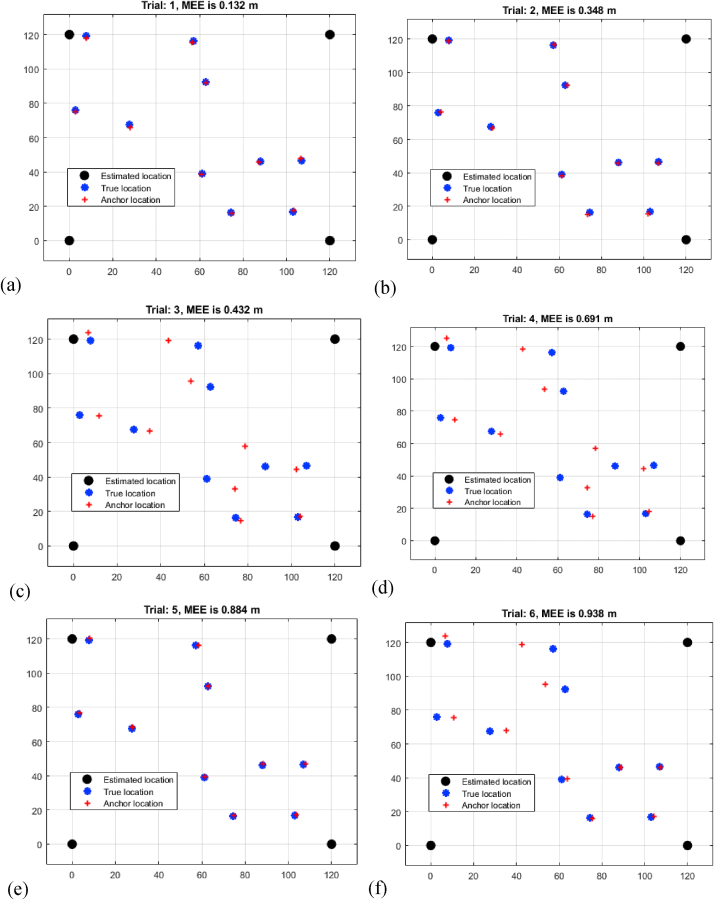

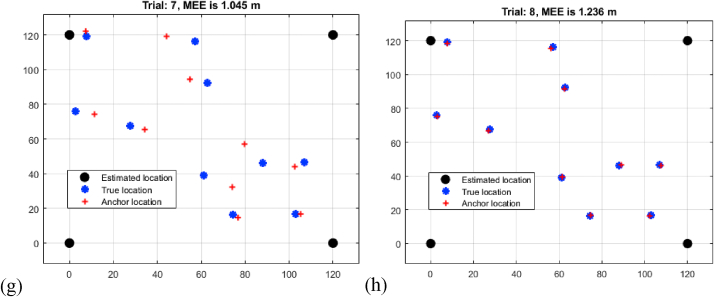
Multi-iteration RNN RSSI mean estimation error analysis (a) trial 1 (b) trial 2 (c) trial 3 (d) trial 4 (e) trial 5 (f) trial 6 (g) trial 7 (h) trial 8.

Table 3, labelled "Multi-Iteration RNN RSSI Mean Estimation Error Analysis," provides substantial insights into the Mean Estimation Errors (MEEs) coming from the application of Recurrent Neural Networks (RNN) to RSSI observations across eight trials of varied distances. As the distance between sensors grows, the results show a distinct pattern in MEEs. Trial 1 has the lowest MEE of 0.132 m over a shorter distance, while Trial 8 has the greatest MEE of 1.236 m over a longer distance shown in Fig. 6(a–h). The results show that the RNN-based technique is effective in mitigating the effects of signal attenuation and environmental fluctuations on RSSI measurements. The progressive increase in MEEs with distance implies that the RNN's capacity to capture temporal dependencies contributes to higher accuracy as compared to conventional RSSI approaches. This trend emphasizes the RNN's ability to compensate for signal fluctuations and improve the reliability of RSSI-based localization over wide ranges. The findings advocate for the use of advanced machine learning algorithms, such as RNNs, to improve the resilience and precision of RSSI-based underwater target localization.

M. Relative analysis

**Table 3:** Multi-iteration RNN RSSI mean estimation error analysis.

Trial	Distance (m)
1	0.132
2	0.348
3	0.432
4	0.691
5	0.884
6	0.938
7	1.045
8	1.236

Results from the study "Enhancing Underwater Target Localization through Proximity-Driven Recurrent Neural Networks" showed a substantial enhancement in the accuracy of localizing targets beneath the water. When applied to underwater environments, the proposed Proximity-Driven Recurrent Neural Networks (PD-RNN) method outperforms prior approaches and considerably reduces the difficulties created by the complexity of the surrounding environment. Accounting for sensor flaws and uncertainties natural to underwater environments, the PD-RNN efficiently boosts accuracy by incorporating information from nearby sensors or targets. The method performs exceptionally well in a variety of conditions, such as those with varying water depths, target mobility levels, and the presence of obstructions. The PD-RNN also has the added virtue of being computationally efficient, making it a good fit for use in real-time underwater robotics. While the study does have its limitations—for example, in highly crowded environments—it does lay the groundwork for future studies that try to refine and expand the method's use, offering a potential means of significantly enhancing underwater target localization.

**Fig. 7:**
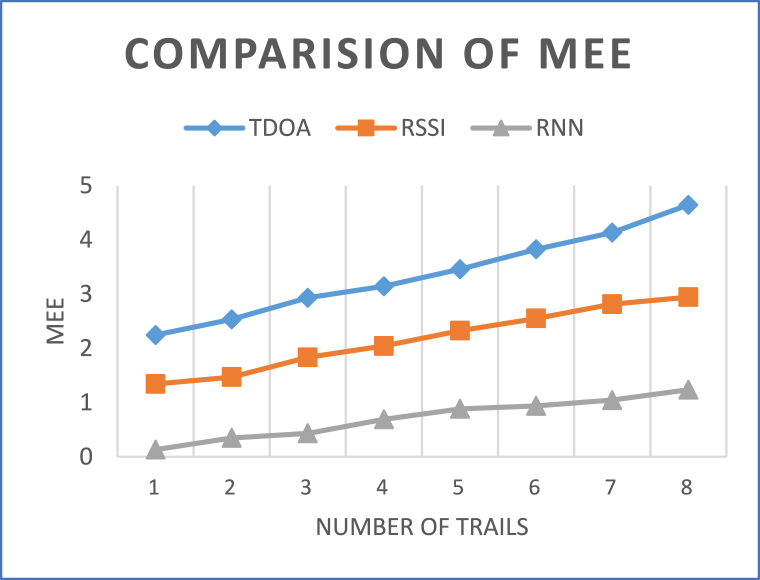
Comparisons of MEEs.

Table 4 titled "Data analysis of MEEs for six iterations," provides critical insights into the Mean Estimation Errors (MEEs) resulting from three distinct localization methods—Time Difference of Arrival (TDOA), Received Signal Strength Indication (RSSI), and RNN-enhanced RSSI (RNN-RSSI)—across eight trials spanning various distances. The results show significant trends across all techniques as shown in Fig. 7. While TDOA has low MEEs over short distances, it exhibits incremental error development over longer distances. RSSI, on the other hand, has bigger beginning mistakes but performs better over extended distances due to its adaptability in handling signal attenuation issues. The unique RNN-RSSI strategy consistently produces low MEEs regardless of distance, demonstrating its promise in combining the strengths of both RSSI and recurrent neural networks to alleviate mistakes caused by distance-related problems. The analysis emphasizes the trade-offs and benefits associated with each approach under various distance scenarios. Importantly, the RNN-RSSI's capacity to retain low MEEs over long distances shows that it can achieve precise underwater target localization even under challenging situations. This comparison improves comprehension of each method's performance characteristics and enables educated decision-making in selecting the most suited technique based on localization needs and environmental limits.

**Table 4:** Data analysis of MEEs for 8 iterations.

Trial	Distance (m)		
	TDOA	RSSI	RNN-RSSI
1	2.245	1.342	0.132
2	2.532	1.468	0.348
3	2.931	1.832	0.432
4	3.142	2.041	0.691
5	3.456	2.324	0.884
6	3.824	2.549	0.938
7	4.132	2.813	1.045
8	4.643	2.942	1.236

Comparing three localization methods—TDOA, RSSI, and RNN-RSSI—based on the presented dataset of distances shows significant performance trends. TDOA has a smaller Mean Estimation Error (MEE) for shorter distances (2.245–3.142 m), but it increases with distance. RSSI, with slightly larger starting mistakes, improves with distance due to its capacity to counter signal attenuation. RNN-RSSI outperforms both techniques at all distances with low MEEs (0.132–1.236 m). This shows how Recurrent Neural Networks can improve RSSI-based localization accuracy by maintaining precision at different distances, overcoming signal variability and distance-induced mistakes.

## 6 Conclusion

To summarize, “Enhancing Underwater Target Localization through Proximity-Driven Recurrent Neural Networks" provides an innovative and efficient method for improving underwater target localization precision. The research reveals that integrating proximity-driven information using Recurrent Neural Networks (RNNs) improves localization accuracy significantly, especially in tough underwater settings. The multi-iteration Angle-based Mean Estimation Errors (MEEs) are thoroughly examined to demonstrate the suggested method's convergence behaviour and consistency, demonstrating its long-term reliability. Furthermore, the study contributes to a more complete picture of underwater target localization by emphasizing the importance of angle-based data alongside traditional distance-based approaches. TDOA has a smaller MEE for shorter distances (2.245–3.142 m), but it increases with distance. RSSI, with slightly larger starting mistakes, improves with distance due to its capacity to counter signal attenuation. RNN-RSSI outperforms both techniques at all distances with low MEEs (0.132–1.236 m). These findings illustrate the potential and utility of the proposed method for practical underwater applications by integrating proximity-driven information with RNNs effectively overcoming the theoretical-to-practical gap, enabling accurate and trustworthy underwater target localization. The findings of the study can be used to improve localization systems and enhance the capabilities of autonomous underwater devices in the future.

## Funding

This study was financially supported by 10.13039/501100002456Chonnam National University (Grant number: 2024-0926).

## Data availability statement

All the data are included in this paper is not deposited in any publicly available repository and will be made available on request.

## CRediT authorship contribution statement

**Sathish Kumar:** Conceptualization. **Ravikumar Chinthaginjala:** Data curation. **Dhanamjayulu C:** Data curation. **Tai-hoon Kim:** Project administration, Funding acquisition. **Mohammed Abbas:** Investigation. **Giovanni Pau:** Supervision. **Nava Bharath Reddy:** Methodology.

## Declaration of competing interest

The authors declare the following financial interests/personal relationships which may be considered as potential competing interestsTai-hoon Kim reports financial support was provided by 10.13039/501100002456Chonnam National University - Yeosu Campus. Tai-hoon Kim reports a relationship with Chonnam National University - Yeosu Campus that includes: employment. If there are other authors, they declare that they have no known competing financial interests or personal relationships that could have appeared to influence the work reported in this paper.

## Abbreviations

(RNNs): Recurrent Neural Networks
(MEEs): Mean Estimation Errors
(UWSNs): Underwater wireless sensor networks
(Cogni Loc): Cognitive Localization
(TPENN): Temporal profit Elman neural network
(MPC): Model predictive controller
(DRL): Deep reinforcement learning
(LSTM-NN): Long-term and short-term memory
(CNN): Convolutional neural network
(CBF): Conventional beamforming
(MUSIC): Multiple signal classification
(MVDR): Minimum-variance distortion less response
(SBL): Sparse Bayesian learning
(RMSE): Root-mean-squared error
(DCNN): Deep convolutional neural network
(KF): Kalman Filter
(RSSI): Received Signal Strength Indicator
(TDOA): Time Difference of Arrival
(PD-RNN): Proximity-Driven Recurrent Neural Networks

## References

[1] Sathish K., Ravikumar C.V., Rajesh A., Pau G. (2022). Underwater wireless sensor network performance analysis using diverse routing protocols. J. Sens. Actuator Netw..

[2] Rani S., Talwar R., Malhotra J., Ahmed S.H., Sarkar M., Song H. (2015). A novel scheme for an energy-efficient Internet of Things based on wireless sensor networks. Sensors.

[3] Heidemann J., Ye W., Wills J., Syed A., Li Y. (2006). Wireless Communications and Networking Conference (WCNC 2006).

[4] Ravikumar C.V., Praveen Bagadi Kala (2019). Design of MC-CDMA receiver using RBF network to mitigate MAI and nonlinear distortion. Neural Comput. Appl..

[5] Chuku N., Nasipuri A. (2021). RSSI-based localization schemes for wireless sensor networks using outlier detection. J. Sens. Actuator Netw..

[6] Park M.K., Rodoplu V. (2007). UWAN-MAC: an energy-efficient MAC protocol for underwater acoustic wireless sensor networks. IEEE J. Ocean. Eng..

[7] Sathish et al. (2023). Underwater wireless sensor networks with RSSI-based advanced efficiency-driven localization and unprecedented accuracy. Sensors.

[8] Srinivasulu Asadi (2022). Performance and improvement analysis of the underwater WSN using a diverse routing protocol approach. Journal of Computer Networks and Communications.

[9] Liu L., Zhou Z., Han G., Martínez-García M. (2022). IEEE Transactions on Mobile Computing.

[10] Saeed K., Khalil W., Al-Shamayleh A.S., Ahmad I., Akhunzada A., Alharethi S.Z., Gani A. (2023). Analyzing the impact of active attack on the performance of the AMCTD protocol in underwater wireless sensor networks. Sensors.

[11] Bhattacharjya K., Alam S., De D. (2018). 2nd International Conference on Computational Intelligence, Communications and Business Analytics (CICBA).

[12] Alkindi Z., Alzeidi N., Touzene B.A.A. (2018). Performance evolution of grid-based routing protocol for underwater wireless sensor networks under different mobile models. Int. J. Wireless Mobile Network.

[13] Fang Z., Wang J., Du J., Hou X., Ren Y., Han Z. (2022). Stochastic optimization-aided energy-efficient information collection in internet of underwater things networks. IEEE Internet Things J..

[14] Bagadi K. (2022). Detection of signals in MC–CDMA using a novel iterative block decision feedback equalizer. IEEE Access.

[15] Venkata R.C. (2023). Review of localization and clustering in USV and AUV for underwater wireless sensor networks. Tele.com (NY).

[16] Xu T., Wang J., Shi W., Wang J., Chen Z. (2019). A localization algorithm using a mobile anchor node based on region determination in underwater wireless sensor networks. J. Ocean Univ. China.

[17] Hamdi M. (2023). Reliable data transmission in underwater wireless sensor networks using a cluster-based routing protocol endorsed by member nodes. Electronics.

[18] Yan J., Li X., Lu X., Guan X. (2017). Virtual-lattice based intrusion detection algorithm over actuator-assisted underwater wireless sensor networks. Sensors.

[19] Cao X., Ren L., Sun C. (2022). Dynamic target tracking control of autonomous underwater vehicle based on trajectory prediction. IEEE Trans. Cybern..

[20] Song D., Gan W., Yao P., Zang W., Zhang Z., Qu X. (2022). Guidance and control of autonomous surface underwater vehicles for target tracking in ocean environment by deep reinforcement learning. Ocean Eng..

[21] Wang M., Xu C., Zhou C., Gong Y., Qiu B. (2022). Study on underwater target tracking technology based on an LSTM–Kalman filtering method. Appl. Sci..

[22] Shin W., Kim D.S., Ko H. (2023). Target tracking from weak acoustic signals in an underwater environment using a deep segmentation network. J. Mar. Sci. Eng..

[23] Nie W., Zhang X., Xu J., Guo L., Yan Y. (2023). Adaptive direction-of-arrival estimation using deep neural network in marine acoustic environment. IEEE Sensor. J..

[24] He S., Li Q., Khishe M., Salih Mohammed A., Mohammadi H., Mohammadi M. (2023). The optimization of nodes clustering and multi-hop routing protocol using hierarchical chimp optimization for sustainable energy efficient underwater wireless sensor networks. Wireless Network.

[25] Yang Y., Wu Y., Yuan H., Khishe M., Mohammadi M. (2022). Nodes clustering and multi-hop routing protocol optimization using hybrid chimp optimization and hunger games search algorithms for sustainable energy efficient underwater wireless sensor networks. Sustainable Computing: Informatics and Systems.

[26] Khishe M. (2022). Drw-ae: a deep recurrent-wavelet autoencoder for underwater target recognition. IEEE J. Ocean. Eng..

[27] Khishe M., Mohammadi M., Rashid T.A., Mahmud H., Mirjalili S. (2022). Handbook of Moth-Flame Optimization Algorithm.

[28] Khishe M., Mohammadi M., Ramezani Varkani A. (2023). Underwater backscatter recognition using deep fuzzy extreme convolutional neural network optimized via hunger games search. Neural Process. Lett..

[29] Kamalipour M., Agahi H., Khishe M., Mahmoodzadeh A. (2022). Variable-length deep convolutional neural networks by internet protocol chimp optimization algorithm for underwater micro-target classification. Iranian journal of Marine technology.

[30] Sreekala K., Raj N.N., Gupta S., Anitha G., Nanda A.K., Chaturvedi A. (2023). Deep convolutional neural network with Kalman filter based objected tracking and detection in underwater communications. Wireless Network.

[31] Han G.J., Zhang C.Y., Liu T.Q. (2016). A multi-anchor nodes collaborative localization algorithm for underwater acoustic sensor networks. Wireless Commun. Mobile Comput..

[32] Zhou R., Chen J., Tan W., Cai C. (2022). Sensor selection for optimal target localization with 3-D angle of arrival estimation in underwater wireless sensor networks. J. Mar. Sci. Eng..

[33] Li D., Du J., Liu L. (2017). A data forwarding algorithm based on Markov thought in underwater wireless sensor networks. International Journal of Distributed Sensor Ne works.

[34] Anbazhagan R. (2022). Investigation and numerical simulation of the acoustic target strength of the underwater submarine vehicle. Inventions.

[35] Li S., Sun H., Esmaiel H. (2020). Underwater TDOA acoustical location based on majorization-minimization optimization. Sensors.

[36] Arbula D., Ljubic S. (2020). Indoor localization based on infrared angle of arrival sensor network. Sensors.

[37] Ullah Inam (2019). Efficient and accurate target localization in underwater environment. IEEE Access.

[38] Sahota H., Kumar R. (2021). Sensor localization using time of arrival measurements in a multi-media and multi-path application of in-situ wireless soil sensing. Inventions.

[39] Haque K.F., Kabir K.H., Abdelgawad A. (2020). Advancement of routing protocols and applications of underwater wireless sensor network (UWSN)—a survey. J. Sens. Actuator Netw..

[40] Kaveripakam S., Chinthaginjala R. (2023 Aug 15). Energy balanced reliable and effective clustering for underwater wireless sensor networks. Alex. Eng. J..

[41] Dubrovinskaya E., Kebkal V., Kebkal O., Kebkal K., Casari P. (2020). Underwater localization via wideband direction-of-arrival estimation using acoustic arrays of arbitrary shape. Sensors.

[42] Kaveripakam et.al. (2023). Optimal path selection and secured data transmission in underwater acoustic sensor networks: LSTM-based energy prediction. PLoS One.

[43] Sathish et.al. (2023). Clustering-based dragonfly optimization algorithm for underwater wireless sensor networks. Alex. Eng. J..

[44] Rajesh A., Mohammad A., Bal V., Salahuddin K., Giovanni P., See C.H., Iyad D., Livreri P., Abd-Alhameed R. (2023). Enhancement of precise underwater object localization. Radio Sci..

